# Contaminant film thickness affects walkway friction measurements

**DOI:** 10.3389/fpubh.2022.915140

**Published:** 2022-08-30

**Authors:** Dennis D. Chimich, Loay Al-Salehi, Benjamin S. Elkin, Gunter P. Siegmund

**Affiliations:** ^1^MEA Forensic Engineers & Scientists, Richmond, BC, Canada; ^2^Department of Mechanical Engineering, University of British Columbia, Vancouver, BC, Canada; ^3^MEA Forensic Engineers & Scientists, Toronto, ON, Canada; ^4^School of Kinesiology, University of British Columbia, Vancouver, BC, Canada

**Keywords:** surface, slip resistance, available friction, tribometer, slip and fall, contaminant, film thickness

## Abstract

Walkway tribometers are used to measure available friction for evaluating walkway safety and pedestrian slip risk. Numerous variables can affect tribometer measurements, including the type and distribution of contaminants on the surface. Here, we quantified the effect of application method on contaminant film thickness, and the effect of film thickness on tribometer measurements on the four reference walkway surfaces used in ASTM F2508-16e. Distilled water, 0.05% sodium lauryl sulfate (SLS) solution, and 0.04% Triton X-100 solution were poured, squirted, and sprayed onto the surfaces to quantify their naturally occurring film thicknesses. These application methods had a significant effect on the resulting film thickness (*p* < 0.038), with the pour method consistently generating the thickest films and the spray method generating the thinnest films. We then quantified the effect of film thickness for the three contaminants (thickness range 0.3–3.3 mm) on the friction measurements of three common tribometers (Mark IIIB, English XL, and BOT 3000E) on each reference surface. A separate ANOVA was used for each of the 3 × 4 × 3 = 36 combinations of tribometer, surface, and contaminant. Friction measured with the Mark IIIB decreased with increasing film thickness on one surface across all three contaminants and on a second surface with the SLS contaminant. Friction measured with the BOT 3000E was sensitive to film thickness on two surfaces with water and one surface with Triton. The XL was unaffected by contaminant film thickness. Overall, despite significant differences in film thickness with contaminant application method, friction measurements were either insensitive to film thickness or varied only a small amount in all cases except for the Mark IIIB on the roughest surface. Film thickness did not alter the relative slip resistance of the four ASTM F2508 reference surfaces.

## Introduction

Validated walkway tribometers are valuable tools for evaluating the slip resistance of walkway surfaces and improving pedestrian safety (1–4). A tribometer is considered validated if it ranks and differentiates the slip resistance of four reference surfaces in the same order as human subjects did in walking trials (1, 2, 5). Using a validated tribometer, field measurements on a different *in-situ* walking surface can potentially be compared to measurements on the reference surfaces to help quantify the *in-situ* surface's slipperiness. Performance standards also rely on validated tribometer measurements to characterize and classify walkway surfaces (6); therefore, a detailed understanding of the factors that affect walkway friction measurements is crucial to improve pedestrian safety.

Tribometer measurements can vary depending on tribometer model, operator/user, time between tests, test foot, and the presence or absence of a contaminant (1, 2, 7–9). For liquid contaminants, the type and concentration of the contaminant further affect surface slipperiness and slip risk (10–12). The amount of liquid contaminant—especially its thickness underfoot—may also affect surface slipperiness. Proctor and Coleman (13) used fluid dynamic theory to show that film thickness was a major contributor to friction between two surfaces. More specific to the shoe–floor interface, Beschorner et al. (4) found lower coefficients of friction with greater contaminant film thicknesses in robotically driven whole-shoe experiments examining the effects of normal force, speed, and shoe angle on the coefficient of friction.

From a fluid lubrication perspective, a liquid film between two sliding surfaces being pressed together becomes pressurized, which causes the surfaces to stay separated and the coefficient of friction to decrease. Fluid pressures can develop due to both a wedge effect (3, 13–16) and a squeeze-film effect (3, 15, 17, 18). The wedge effect captures how fluid pressure varies with sliding velocity and fluid viscosity, whereas the squeeze-film effect captures how fluid pressure diminishes over time. In addition to these pressure-related effects, the presence of a liquid film on a surface can mask the surface texture and reduce the friction generated by adhesion. Adhesion is particularly sensitive to contaminant viscosity, with higher viscosity fluids causing a greater reduction in adhesion and lower friction (10, 11, 19). These physical phenomena affect the shoe–floor friction during walking (or other human gait-related activities) and likely also affect the friction measurements made using tribometers. Although the research cited above has examined the effect of film thickness on shoe–floor friction during shoe–contaminant–floor interaction, the effect of the initial film thickness of a liquid contaminant on tribometer measurements of friction has not been systematically explored.

The aim of this study was to quantify the effect of the initial contaminant film thickness, measured prior to tribometer test foot contact with the contaminant, on the friction measurements made using walkway tribometers. Since tribometer users apply the contaminant prior to testing, we first evaluated the effect of different application methods on film thickness (herein called the natural film thickness). We then examined the effect of contaminant film thickness that spanned these natural film thicknesses on the friction measurements made using three portable walkway tribometers.

## Methods

### Tribometers and surfaces

**Figure 1:**
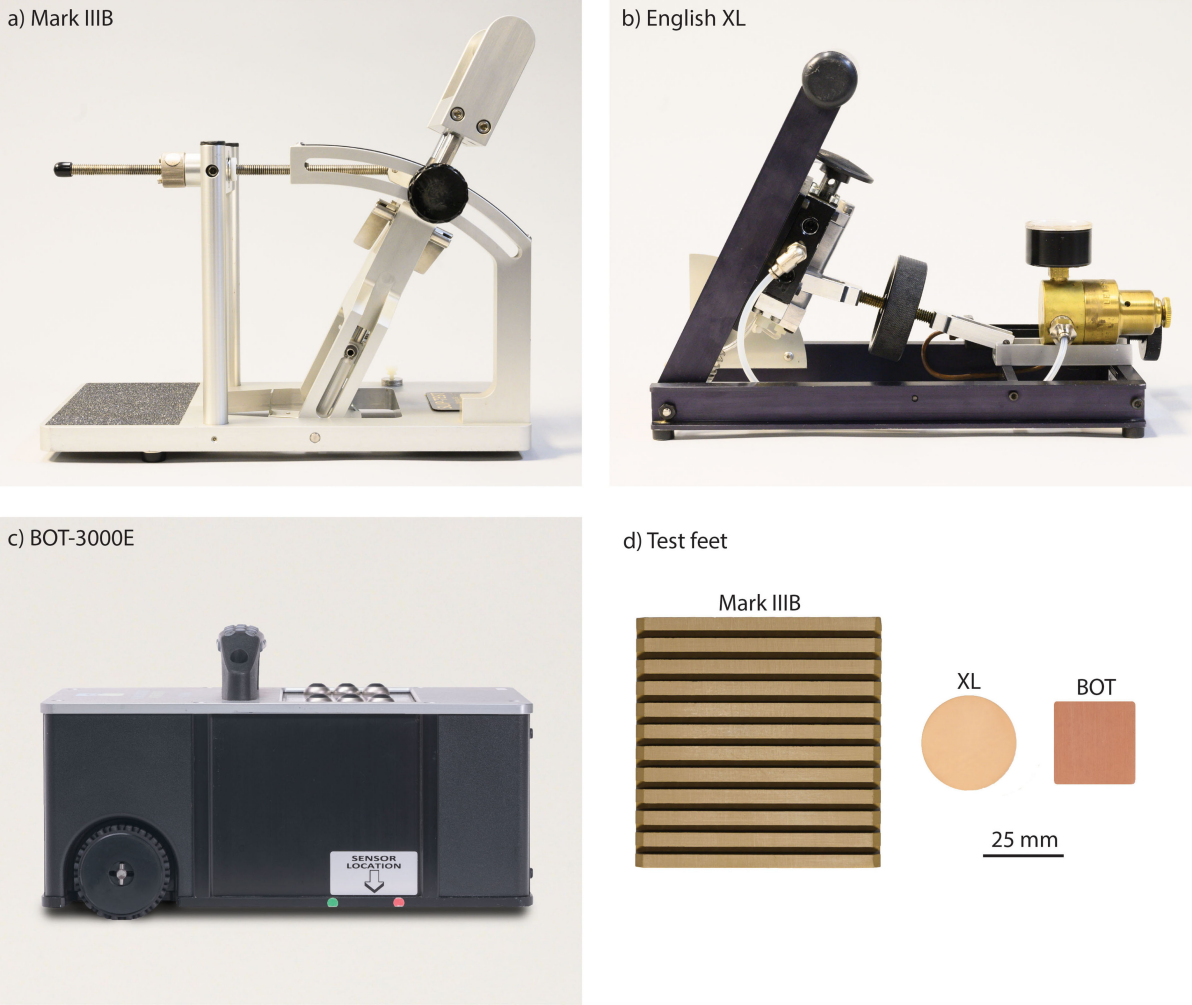
Walkway tribometers tested: **(a)** Mark IIIB, **(b)** English XL, and **(c)** BOT 3000E. **(d)** The test feet for the Mark IIIB and English XL were surfaced with Neolite^Ⓡ^, and the BOT test foot was surfaced with styrene–butadiene–rubber (SBR). The scale applies only to the panel **(d)**.

Three walkway tribometers were used in this study: a Mark IIIB (Slip-Test Inc., Atlanta, GA), an English XL with sequencer (Excel Tribometers LLC, Chesapeake, VA), and a BOT 3000E (Regan Scientific Instruments Inc., Carrollton, TX) (Figure 1). The Mark IIIB test foot (75 mm × 75 mm) was flat, made of Neolite^Ⓡ^, and had 15 evenly spaced grooves (1 mm wide) that ran parallel to the slip direction. The XL test foot (31.5 mm diameter) was also flat and made of Neolite^Ⓡ^, but had no grooves. The BOT test foot/slider (27.8 mm × 27.8 mm) was cylindrical, made of styrene–butadiene–rubber (SBR), and also had no grooves. Based on five measurements taken across the surface of each test foot, the average hardness of each test foot was 95.8 ± 0.45 (Mark IIIB), 96.6 ± 0.55 (English XL), and 97.8 ± 0.45 (BOT 3000E) (Type A Durometer, Model 306L, PTC Instruments, Los Angeles, CA).

All the three tribometer models had published validation data within 5 years of these tests, and each tribometer unit used in the study was calibrated by the user within the last year according to ASTM F2508-16e1 (5). Two of the tribometers (Mark IIIB and BOT 3000E) had published precision statements in accordance with ASTM E691 (20) within the last 5 years.

**Figure 2:**
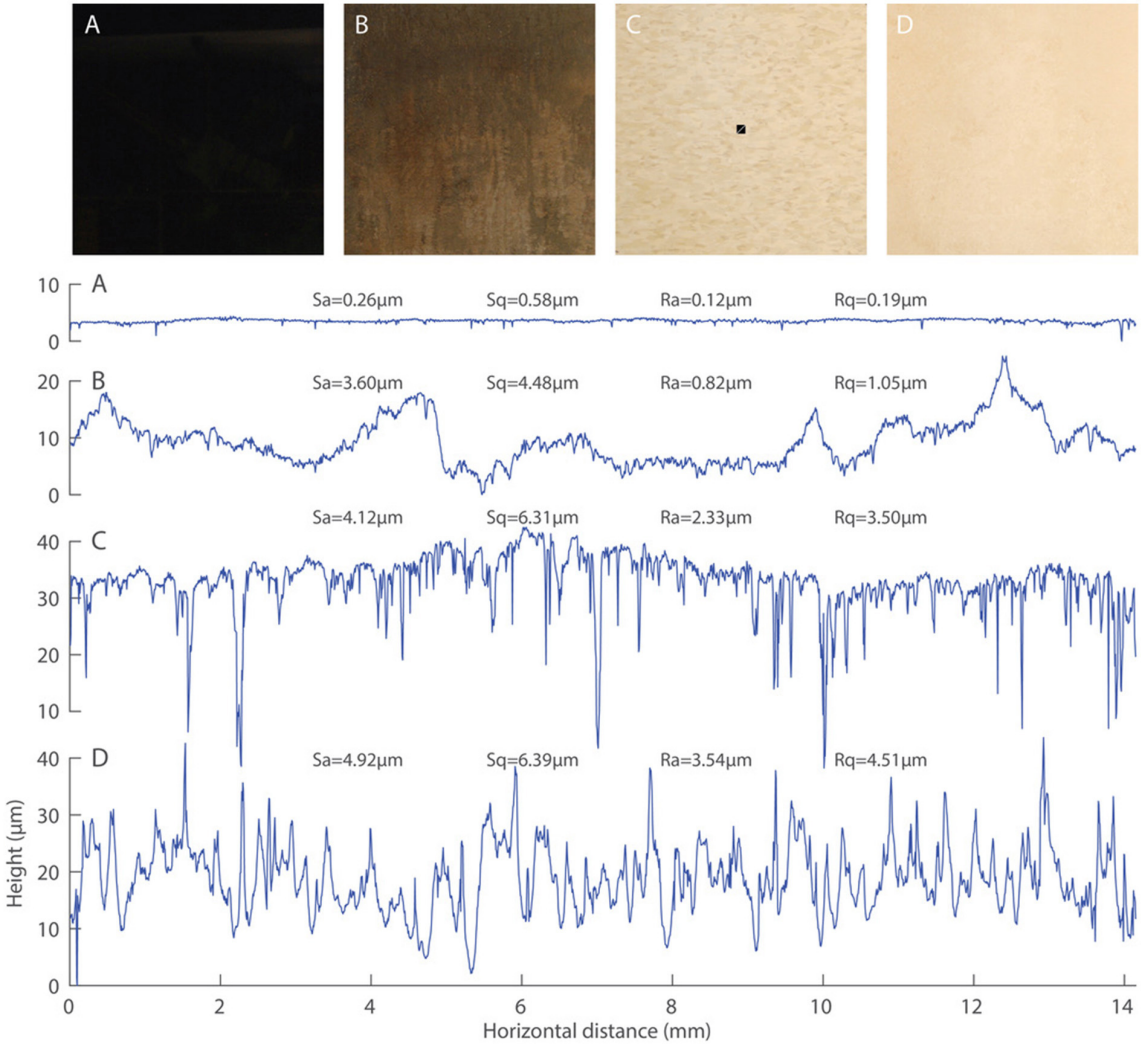
Four reference surfaces adopted by ASTM F2508 and used for all testing: **(A)** Granite. **(B)** Porcelain. **(C)** Vinyl composition tile (VCT). **(D)** Ceramic. All surfaces were nominally 30.5 × 30.5 cm (12 × 12 in). Below the four surfaces are representative surface profiles along the 14.04-mm diagonal of a 10 mm × 10 mm square centered on each surface (e.g., see the diagonal white line within the black square centered on surface **(C)**. Also shown are height and roughness parameters for each surface: Sa, arithmetic-mean-height; Sq, root-mean-square height; Ra, arithmetic-mean-deviation; Rq, root-mean-square deviation.

All testing was performed on four reference surfaces (A: granite, B: porcelain, C: vinyl composition tile [VCT], and D: ceramic) that prior human subject tests have shown possess different slip risks when contaminated by a poured fluid film of undocumented thickness (1) (Figure 2). These four surfaces have been adopted by ASTM F2508 for calibration, validation, and certification of walkway tribometers. The roughness of each surface was measured using a 3D non-contact profilometer (ST400, Nanovea, Irvine, CA) using an LS1 optical pen over a 10 mm × 10 mm area at the center of each surface. Two height parameters (Sa and Sq) were calculated over the entire scanned area according to ISO 25178 (21), and two roughness parameters (Ra and Rq) were calculated along the diagonal of the scanned area according to ISO 4287 (22) (Figure 2). All reference surfaces were cleaned according to ASTM F2508 prior to natural film thickness and friction testing. None of the surfaces showed visible wear or deterioration before or after this study.

### Test procedures

#### Natural film thickness

The natural film thickness was quantified for three application methods on each of the four reference surfaces using three contaminants. The tile surfaces were placed on a level concrete table and sufficient contaminant was then applied to cover a 10 cm × 10 cm area centered on the tile. A continuous film suitable for slip testing was achieved by pouring each contaminant from a 500-ml graduated cylinder, squirting from a 500-ml wash bottle with an angled nozzle, or spraying from a 500-ml household spray bottle. The resulting film thickness was measured at the center of the designated area using a F70 Thin-Film Analyzer (Filmetrics Inc., San Diego, CA). The three contaminants were distilled water, a 0.05% solution of SLS, and a 0.04% solution of Triton X-100. The viscosity and density of each contaminant was determined using ASTM D1217 (23) and ASTM D445 (24), respectively, and varied by less than 1% between contaminants (Table 1). The 12 combinations of four reference surfaces and three contaminants were tested in random order. Within each combination, each application method was repeated 10 times with the order of all 30 applications (10 per method) randomized. All 360 tests (4 reference surfaces × 3 application methods × 3 contaminants × 10 repetitions) were performed by the same user (author DC). Prior to each surface/contaminant combination, the surfaces were cleaned according to the ASTM 2508-16 standard. Before the initial contaminant application on a surface, the surface was cleaned with 50% ethanol with a clean towel and the surface was air-dried. Between applications, the puddle from the prior test was removed with a towel, the surface was cleaned with 50% ethanol using a separate clean towel, and the surface was air-dried.

**Table 1:** Density and viscosity of the contaminants.

**Contaminant**	**Density (kg/L)**	**Viscosity (cSt)**
Distilled water	1.000	1.02
0.04% triton	1.001	1.01
0.05% SLS	1.000	1.02

cSt = mm^2^/s.

#### Film thickness effect

The effect of film thickness on slip resistance measurements was assessed for all combinations of the three tribometers (Figure 1), four reference surfaces (Figure 2), and three contaminants. All surfaces were cleaned before each test series, placed on a level concrete table and tested in the same direction throughout. To repeatedly create specific film thicknesses, a custom reservoir, made using adhesive aluminum tape (Cantech, Montreal QC) adhered around the perimeter of a reference surface, was utilized for the Mark IIIB and English XL tests. For the BOT 3000E, a narrow metal frame was attached to the tile using a double-sided, non-cured adhesive rubber transfer tape (Transfer Tape 0485, size: 1/25″ thick × 1/2″ wide, Scapa Group, Rotunda, UK). Foam rubber tape made of neoprene (CF21001 1/8″ thick × 3/8″ wide, RCR, Mississauga, ON) was placed on the lower side of the metal frame to deepen the reservoir and to prevent leakage. The metal frame reservoir fit under the BOT 3000E and between the wheels so that the wheels did not get wet during testing. Three contaminant thicknesses of nominally 1.3, 2.3, and 3.3 mm were used for each combination of tribometer, surface, and contaminant. For all combinations, a fourth and sometimes fifth thickness were added near the natural film thickness observed in the prior experiment. The contaminant was applied using the wash bottle until the desired depth, as measured by the Thin-Film Analyzer, was achieved. Each tribometer's test foot was prepared according to its manufacturer's instructions (25–27). For the Mark IIIB, the test foot was sanded 4 times along the grooves and 4 times perpendicular to the grooves; five conditioning slips were then performed on the surface prior to testing. For the English XL, the test foot was sanded using The sander and calibrated on the manufacturer's calibration tile prior to testing and following every eight slips with a slip resistance above 0.20. At least 2 non-slips were performed prior to each slip measurement. For the BOT, the test foot was sanded with a sanding pad and/or the sensor reconditioning/sanding tool before testing and after every 5 trials, and then tested on the supplied reference surface before continuing. All testing for a specific combination of tribometer and surface was completed in a single block. For the Mark IIIB and XL, six slip resistance measurements were made at each contaminant thickness within each block (thickness order randomized). For the BOT 3000E, two series of five measurements were made at each contaminant thickness to accommodate the sanding protocol. The thickness within each series was constant, but the thickness order between series was randomized.

### Statistical analysis

#### Natural film thickness

To assess the effect of application method on natural film thickness, we used a general linear mixed model (GLMM) with thickness as the response variable and application method (pour, squirt, and spray) and reference surface (A, B, C, and D) as the predictor variables. A separate GLMM was run for each contaminant. We included a fixed intercept and slope for the application method, and a random intercept and slope for the application method grouped by tile (see Equation 1, in Wilkinson notation). The mixed model analysis was run using the *fitlme* subroutine in MATLAB (2021b, MathWorks, Natick, MA) using maximum likelihood (ML) estimation, the Satterthwaite approximation for degrees of freedom, and a significance level of α <0.05.


(1) $$\begin{array}{r} Thickness\sim 1+Method+\left(1+Method|Surface\right) \end{array}$$

#### Film thickness effect

To assess the effect of film thickness on measured friction, we used a separate one-way analysis of variance (ANOVA) for each combination of tribometer, surface, and contaminant (*n* = 36 analyses). The use of different film thicknesses for some combinations of tribometer, surface, and contaminant prevented a combined multiway analysis. *Post-hoc* comparisons between different thicknesses were performed using Tukey's honest significant difference test. All tests were performed using the *anova1* subroutine in MATLAB. A significance level of α <0.05 was used for all tests. The significance level was not adjusted for multiple comparisons because the number of tribometers, contaminants, and surfaces we chose to test should not affect the probability of identifying a particular combination for which friction varies with contaminant film thickness.

## Results

### Natural film thickness

**Figure 3:**
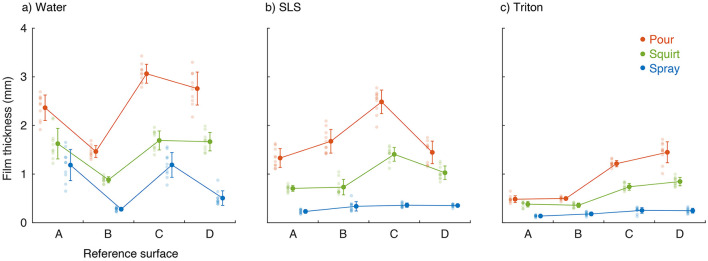
Raw data and mean ± SD of the natural film thickness for the three application methods (pour, squirt, and spray) on the four reference surfaces (A, B, C, D) for the three contaminants: **(a)** distilled water, **(b)** 0.05% SLS solution, and **(c)** 0.04% Triton solution.

Across all surfaces and contaminants, the pour application method consistently yielded the thickest films, and the spray application method consistently yielded the thinnest films (Figure 3). The squirt method was on average 0.95, 0.77, and 0.33 mm thinner than the pour method for the water, SLS, and Triton contaminants, respectively. The spray method was on average 1.62, 1.41, and 0.71 mm thinner than the pour method for the water, SLS, and Triton contaminants, respectively. Only minor surface-specific effects were observed. For water on surface B, the film thickness across all three application methods was on average 0.95 mm thinner than on the other three surfaces, which were not different from each other. For SLS on surface C, the pour method was 0.74 mm thicker, and the squirt method was 0.43 mm thicker than on the other three surfaces, which were not significantly different from each other. A summary of the statistical output is given in Supplementary Tables S1–S6.

### Film thickness effect

**Figure 4:**
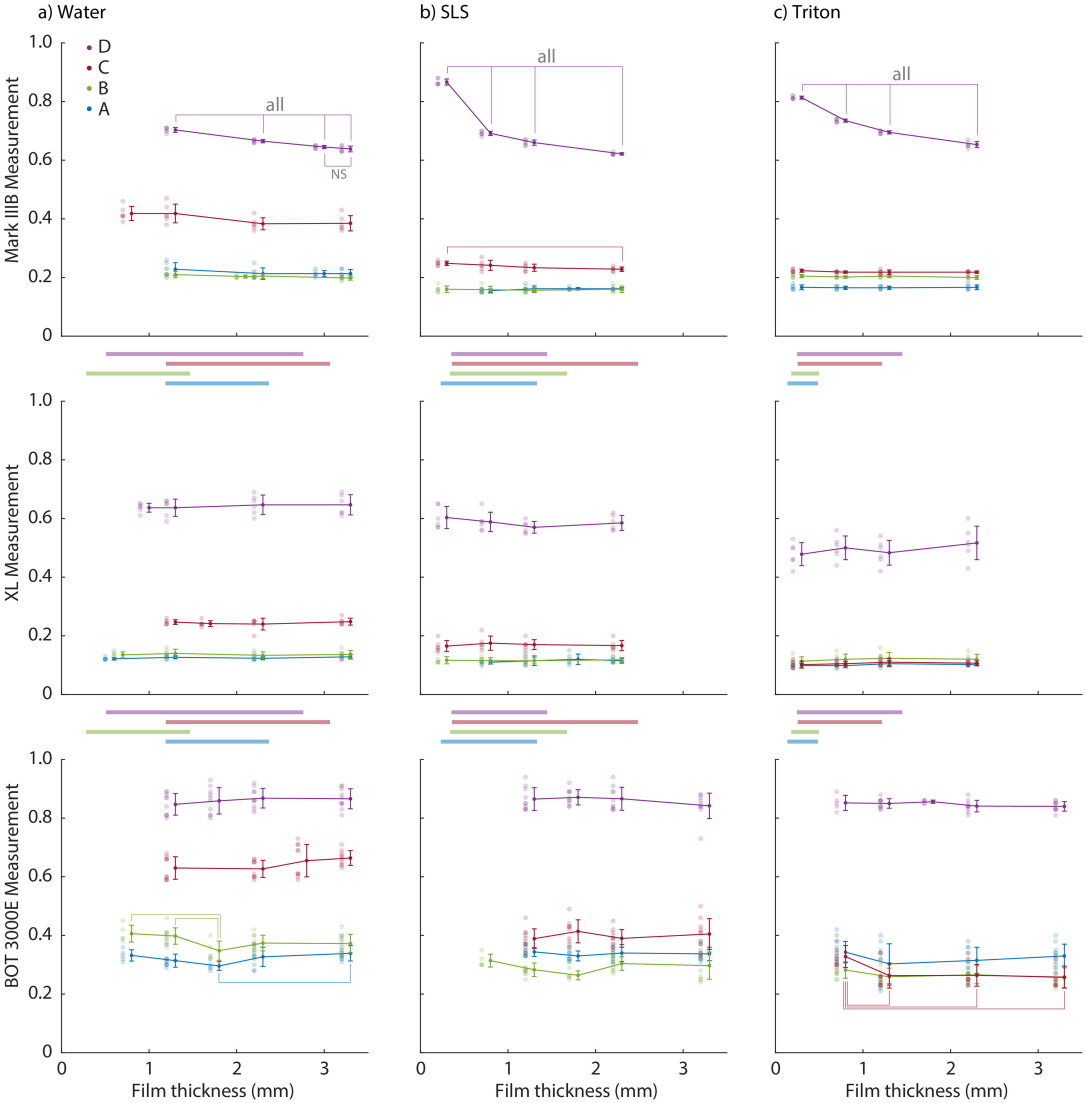
Raw data and mean ± SD of the friction measurements as a function of film thickness for the four reference surfaces (colored lines within each graph), three tribometer models (rows of graphs) and three contaminants [columns of graphs: **(a)** water, **(b)** SLS, and **(c)** Triton]. Significant differences within each combination of tribometer, surface, and contaminant are highlighted using thin vertical and horizontal bars; “all” means that all connected comparisons are significantly different (unless otherwise noted). The thick horizontal bars between the rows of graphs show the range of natural film thicknesses for each combination of contaminant and surface (colors match the surfaces in the graphs).

Overall, contaminant film thickness affected friction measurements in 7 of the 36 combinations of tribometer, surface, and contaminant we tested (Figure 4). Film thickness affected the slip resistance measured by the Mark IIIB on surface D for all three contaminants, where aside from the two thickest water films, each incremental increase in film thickness was significantly slipperier. For SLS on surface C with the Mark IIIB, the thickest film (3.3 mm) was also more slippery than the thinnest film (0.3 mm). For the BOT 3000E, the effects of film thickness were more variable. Surface A was slipperier with 3.3 mm of water than with 1.8 mm of water, and surface B was slipperier with 1.8 mm of water than with both 0.8 and 1.3 mm of water. Surface C was less slippery with 0.8 mm of the Triton solution than with 1.3, 2.3, and 3.3 mm of the Triton solution. In contrast to both the Mark IIIB and the BOT 3000E, the English XL was insensitive to film thickness on all four surfaces with all three contaminants.

## Discussion

Our aim was first to quantify the natural film thickness of three contaminants applied to four reference surfaces and then to quantify the effect of contaminant film thickness on the friction measurements made using three common walkway tribometers. Overall, we found that the application method had a large and consistent effect on contaminant natural film thickness, but that film thickness had a smaller and more variable effect on friction measurement that was tribometer and surface-specific. These findings have important implications for both safety and the investigation of slip-and-fall incidents.

### Natural film thickness

We found that poured films were thicker than squirted films, and squirted films were thicker than sprayed films. For the 12 combinations of surfaces and contaminants, squirted film thickness was about 62 ± 9% (range 46–76%) of poured film thickness, and sprayed film thickness was about 25 ± 10% (range 15–49%) of the poured film thickness. One explanation for this consistent pattern may be differences in the user's ability to accurately regulate the amount of fluid applied by each method. For instance, the graduated cylinder had an open top and required the user to control a shifting center of mass as the cylinder and contaminant were simultaneously tilted to pour the liquid onto the surface. The wash bottle, by contrast, remained vertical, and the volume of expelled fluid was controlled by squeezing the soft bottle while the fluid stream could be directed to areas of the surface that required more fluid. The spray bottle further increased the user's control by expelling only small amounts of fluid with each pull of the lever while simultaneously distributing this smaller volume over a wide area. The distribution of the raw data in Figure 3 also revealed lower variability for the spray method, especially for SLS and Triton and suggests that the spray application method is preferable over the other two methods when thin and repeatable film thicknesses are desired.

The natural film thickness we observed with the contaminants containing detergents (0.05% SLS and 0.04% Triton X-100) was thinner than the films we observed with distilled water (*post-hoc* paired *t*-tests, *p* < 0.002). This phenomenon may be partially explained by the lower surface tension created by the addition of detergents or surfactants to water. Surfactants lower the surface tension by separating the water molecules from one another. Despite this overall pattern of thinner films with the detergent contaminants, the film created on surface B (porcelain) with SLS was slightly thicker than with water for both the pour and spray methods. This counterintuitive result shows that film thickness is not solely a function of surface tension, and that other factors related to the surface may also play a role. Indeed, natural film thickness is also affected by the spreading coefficient of a liquid, which is the measure of the tendency of a liquid to spread on a second phase, in this case the solid tile surface (28).

### Film thickness effect

In contrast to our consistent natural film thickness findings, a significant effect of initial film thickness on friction was isolated to only a few combinations of the tribometers, surfaces, and contaminants tested here. The largest absolute effect of film thickness occurred with the Mark IIIB on surface D, where thicker films yielded lower levels of friction for all three contaminants (top row, Figure 4). A similar, albeit attenuated, pattern was also seen for the Mark IIIB on surface C with SLS, but was absent for the remaining combinations of surfaces and contaminants for the Mark IIIB and for all combinations of surfaces and contaminants for the XL. Reduced friction with thicker films is consistent with squeeze-film theory, in which the time (*t*) to reduce a film from an initial thickness (*h*_0_) to a final thickness (*h*) is shown below (29):


$$\begin{array}{l} t=\frac{K\eta A^{2}}{F_{N}}\left(\frac{1}{h^{2}}-\frac{1}{h_{0}^{2}}\right) \end{array}$$

where *K* is a factor related to the shape of the squeezed area, η is the fluid viscosity, A is the squeezed area, and *F*_*N*_ is the normal force pressing the two surfaces together. For a larger initial film thickness (*h*_0_), the time to squeeze out the film increases, resulting in less interaction between the test foot and the surface during the short duration of a test and thus a lower measured friction. The reason this effect was seen only for the Mark IIIB, and then most strongly on surface D may be related to two interacting factors. First, the area of the Mark IIIB's test foot is large: 7.25 times larger than the area of the XL's test foot. This larger area would result in squeeze times that are 53 times longer (*t* ~ *A*^2^), although this effect would be attenuated by the 2.5-mm-wide and 4-mm-deep grooves in the Mark IIIB's test foot. Increased squeeze times for the Mark IIIB mean that it is more sensitive to increases in the initial film thickness and likely explain why the Mark IIIB was more prone to exhibit a squeeze-film effect than the XL; however, they do not explain why only surface D and, to a lesser extent, surface C exhibited a film thickness effect within the Mark IIIB data. This surface-specific effect may be related to a second factor: surface roughness (10, 12, 29). Surface D was the roughest of the four surfaces tested, and its larger asperities (see profiles in Figure 2) may interact more effectively with the Mark IIIB's test foot with thinner initial film thicknesses. With the addition of increasing amounts of contaminant, different combinations of tribometers (i.e., test foot shape, size, material, normal force, etc.) and surfaces (i.e., material, surface roughness, etc.) would have different transitions from a dry regime, where the adhesion component of friction dominates, to a wet regime, where the hysteresis component of friction dominates, to a thick-film regime, where the viscosity of the contaminant dominates (30). The degree to which this pattern manifests in test data may depend on a specific combination of tribometer, surface, and contaminant properties and provides a plausible explanation for the different surface-specific responses we observed within the Mark IIIB data for the initial film thicknesses tested here.

The operating principle of the BOT is different from the other two tribometers, and its measurement of dynamic friction by dragging a test foot at a fixed speed potentially renders it susceptible to wedge effects described in the following equation [for a rectangular test foot, (13)]:


$$\begin{array}{l} h^{2}=\;\frac{K\eta l^{2}b}{F_{N}}\text{v} \end{array}$$

where *h* is the thickness of the film that develops between the test foot and the surface, *K* is a combination of constants related to the test foot geometry and wedge angle, η is the fluid viscosity, *l* is the length of the test foot, *b* is the width of the test foot, *F*_*N*_ is the normal force, and *v* is the velocity of the test foot. Assuming a fixed test foot geometry, normal force, and test speed, the film thickness that develops between the test foot and the surface is proportional to the square root of the viscosity. The difference in viscosity between the contaminants used here was negligible; therefore, similar film thicknesses between a given test foot/surface combination are expected for all three contaminants. As the film thickness (*h*) at the test foot increases above the surface roughness, the friction would be expected to transition from a regime where hysteresis dominates to a regime where viscosity dominates (30). Since the BOT's test foot is pressed into the fluid before the start of the forward motion, the initial film thickness (*h*_0_) and squeeze-film effects likely play a small role. This contention is supported by earlier observations that the Tortus, a tribometer with a design similar to the BOT, does not capture the effect of aquaplaning on smooth floors (31). Thus, neither wedge effects nor squeeze-film theory appear to explain the initial-film-thickness-related differences in friction we observed in 3 of the 12 combinations of surfaces and contaminants measured using the BOT, and further work is needed to explain these differences.

Aside from surface D, the maximum differences we observed between mean friction levels on the same surface with SLS was 0.02 (Mark IIIB, surface C) and 0.01 (XL, surfaces A and C). These values are smaller than the previously reported repeatability limits for the Mark IIIB (*r* = 0.025–0.087) and XL (0.037–0.079) on four other surfaces (9), which suggests that the magnitude of initial film thickness effects is small, and day-to-day variance and other sources of variance due to tribometer sample, surface sample, and the user have a greater effect on the measured friction than initial film thickness on many surfaces (8, 9).

### Impact to safety professionals

Overall, our findings suggest that initial film thickness has a limited effect on field measurements for walkway friction. The absence of film thickness effects for the XL suggests that XL users need not consider this phenomenon further. Users of the Mark IIIB can be similarly unworried when testing surfaces with lower friction levels, but should consider the effect of initial film thickness on surfaces with higher friction or roughness levels. From a safety perspective, using a thick film will generate conservatively lower friction levels and reduce the possibility of misclassifying a surface as slip-resistant if a thin film was used with the Mark IIIB. Users of the BOT can also be relatively unworried, particularly since the manufacturer's recommended testing protocol of painting the contaminant with a brush will generate an initial thickness that is less than the maximum values used here. Additional work is needed to assess thinner films for the BOT before we can make recommendations for its response in this region. Regardless of the tribometer being used, our work suggests that a prudent practitioner should record their method of contaminant application and attempt to create a consistent film thickness for their tests.

A secondary observation visible in our data is that contaminant type affects the tribometer-measured friction. The friction measured by all three tribometers on Surface C was particularly sensitive to the addition of either SLS or Triton to distilled water (Figure 4). These differences were robust across film thicknesses, suggesting that the effect was due to contaminant type rather than secondary to a film thickness effect. The effect of different types of contaminants on tribometer-measured friction (11, 32) and on the propensity for humans to slip (33) have both been studied separately, but these two phenomena have not been studied together. The contact pressures and geometries of a human foot striking the ground can be different from those created by tribometers (11), and therefore the initial film thickness effects observed here may not translate directly to human slip risk. In some cases, the rank order of two surfaces changed between different contaminants (e.g., surfaces A and B contaminated with water and SLS as measured by the BOT 3000E, Figure 4). This latter phenomenon poses a challenge to practitioners attempting to relate tribometer measurements using one contaminant to slip risk in the presence of another contaminant, and further work is needed to address this topic.

### Limitations

A limitation of the current study is that film thickness effects were not tested over the full range of natural film thicknesses for all combinations of tribometers, contaminants, and surfaces. In particular, the thin natural film thickness associated with SLS and Triton (<0.8 mm) could not be achieved in the testing reservoir used for the BOT 3000E tests; the narrow width of this reservoir (7.7 cm) and the interaction between the contaminant and the reservoir edges prevented thinner film thicknesses from developing. These thin film thicknesses may be more relevant to BOT 3000E testing because the standard protocol requires that a strip of contaminant be applied with a paint brush (27), which typically creates a thin film on the surface. As a result, the effect of film thickness for thin films may be larger than suggested by the data presented here for the BOT 3000E. Another limitation of our work is the small number of tribometers, surfaces, contaminants, and users we tested. Further work is needed to expand the range of these variables, especially given the pronounced film thickness effect observed in one combination of these factors and not the other combinations. In a prior study, we found a marked sequential decline in each series of BOT measurements (8), and a similar pattern was noted in the current study (see Supplementary Figure S1). In this prior work, we discarded the BOT's first measurement after sanding the test foot to reduce the variance of the reported values. Here, we included the first measurements, but a follow-up analysis in which the first test was omitted yielded essentially the same results. Another limitation is that the volume of contaminant was not controlled in the friction tests over the range of film thicknesses tested. It is possible that the volume of contaminant may have affected the measured friction in addition to the film thickness effects observed. Increased fluid inertia and buoyancy effects on measured friction with greater contaminant volume require further investigation. In addition, the film thickness created and used here represents the minimum film thickness the user could achieve and deemed appropriate for tribometer testing. They do not necessarily represent the film thickness that might develop naturally on these surfaces in the field or that existed for an actual slip-and-fall event. Film thickness, partial contaminant coverage, or flow of contaminant over a surface could be factors that contributed to a slip-and-fall event. One may consider testing under these various contaminant conditions but caution should be exercised when these contaminant conditions fall outside the operating guidelines for the tribometer. Lastly, the ranges of test foot hardness and contaminant viscosity used here were small. While others have shown considerable effects of viscosity (16, 34) and hardness (34) on measured friction, the ranges studied by these other researchers were much larger than or outside the range used in the present study. The small ranges of test foot hardness and contaminant viscosity used here do not allow us to attribute any of the observed friction differences solely to these properties. Additionally, although the test foot material, hardness, tread, and profile are not the same across the Mark IIIB, English XL, and BOT 3000E tribometers, friction measurements were only compared within a given tribometer model.

## Conclusion

In conclusion, we found that natural contaminant films were consistently thickest for the pour method and thinnest for the spray method. We recommend the spray method due to its ability to create thinner films and for its lower variability. We also found that film thickness had a limited effect on tribometer friction measurements: the XL appeared to be insensitive to initial film thickness, the Mark IIIB was sensitive to film thickness for a rough surface and behaved in a pattern consistent with squeeze-film theory, and the BOT exhibited only intermittent and weak sensitivity to initial film thickness. In all but one combination of tribometer, surface, and contaminant, the maximum differences due to initial film thickness were within the repeatability limit for two of these devices. Despite these findings, we recommend contaminant application methods should be detailed by tribometer providers to help reduce the uncertainty associated with tribometer friction measurements.

## Data availability statement

The raw data supporting the conclusions of this article will be made available by the authors, without undue reservation.

## Author contributions

DC contributed to study design and testing protocol, performed testing, and manuscript preparation. LA-S performed testing. BE contributed to study design and manuscript preparation. GS contributed to study design, performed the statistical analysis, and manuscript preparation. All authors contributed to the article and approved the submitted version.

## Conflict of interest

Authors DC, BE, and GS are forensic consultants who investigate slip-related injuries and falls. Authors DC and GS are minority shareholders of MEA Forensic, and GS is a director. BE is the Chairman of the ASTM F13 Committee on Pedestrian/Walkway Safety and Footwear, which is the committee responsible for the F-2508-16 standard. The remaining author declares that the research was conducted in the absence of any commercial or financial relationships that could be construed as a potential conflict of interest

## Publisher's note

All claims expressed in this article are solely those of the authors and do not necessarily represent those of their affiliated organizations, or those of the publisher, the editors and the reviewers. Any product that may be evaluated in this article, or claim that may be made by its manufacturer, is not guaranteed or endorsed by the publisher.
