# Supplementary material for: Contaminant film thickness affects walkway friction measurements
Source: Front Public Health. 2022 Aug 30;10:915140. doi: 10.3389/fpubh.2022.915140 (PMC9469017; doi:10.3389/fpubh.2022.915140)
Supplement: Supplementary file 1 [file Table_1.DOCX]

**Supplemental Materials**

Tables S1to S6: Statistical Output - Natural Film Thickness Data

Thick ~ 1 + Method + (1 + Method | Surface)

The Pour method was designated as the reference method.

Table S1. Intercepts and Slopes for Fixed Effects (Water)

|  | Var | Estimate | SE | tStat | DF | pValue | Lower | Upper |
| --- | --- | --- | --- | --- | --- | --- | --- | --- |
|  | Intercept | 2.4125 | 0.3005 | 8.0270 | 4.0000 | **0.0013** | 1.5780 | 3.2469 |
|  | Squirt | -0.9458 | 0.1539 | -6.1464 | 4.0000 | **0.0036** | -1.3730 | -0.5186 |
|  | Spray | -1.6228 | 0.2302 | -7.0498 | 4.0000 | **0.0021** | -2.2619 | -0.9837 |

Table S2. Intercepts and Slopes for Random Effects (Water)

| Level | Var | Estimate | SE | tStat | DF | pValue | Lower | Upper |
| --- | --- | --- | --- | --- | --- | --- | --- | --- |
| A | Intercept | -0.0146 | 0.3040 | -0.0480 | 4.1807 | 0.9640 | -0.8443 | 0.8152 |
| A | Squirt | 0.1217 | 0.1582 | 0.7690 | 4.0935 | 0.4838 | -0.3137 | 0.5570 |
| A | Spray | 0.3970 | 0.2396 | 1.6567 | 4.6322 | 0.1631 | -0.2340 | 1.0279 |
| B | Intercept | -0.9513 | 0.3040 | -3.1297 | 4.1807 | **0.0331** | -1.7811 | -0.1216 |
| B | Squirt | 0.3980 | 0.1582 | 2.5155 | 4.0935 | 0.0643 | -0.0374 | 0.8333 |
| B | Spray | 0.4405 | 0.2396 | 1.8383 | 4.6322 | 0.1300 | -0.1905 | 1.0714 |
| C | Intercept | 0.6164 | 0.3040 | 2.0279 | 4.1807 | 0.1094 | -0.2133 | 1.4462 |
| C | Squirt | -0.3299 | 0.1582 | -2.0853 | 4.0935 | 0.1038 | -0.7653 | 0.1054 |
| C | Spray | -0.2338 | 0.2396 | -0.9758 | 4.6322 | 0.3773 | -0.8648 | 0.3971 |
| D | Intercept | 0.3495 | 0.3040 | 1.1497 | 4.1807 | 0.3118 | -0.4803 | 1.1792 |
| D | Squirt | -0.1897 | 0.1582 | -1.1992 | 4.0935 | 0.2952 | -0.6251 | 0.2456 |
| D | Spray | -0.6036 | 0.2396 | -2.5191 | 4.6322 | 0.0571 | -1.2346 | 0.0273 |

Table S3. Intercepts and Slopes for Fixed Effects (SLS)

|  | Var | Estimate | SE | tStat | DF | pValue | Lower | Upper |
| --- | --- | --- | --- | --- | --- | --- | --- | --- |
|  | Intercept | 1.7338 | 0.2255 | 7.6901 | 4.0000 | **0.0015** | 1.1078 | 2.3598 |
|  | Squirt | -0.7658 | 0.1299 | -5.8948 | 4.0001 | **0.0041** | -1.1264 | -0.4051 |
|  | Spray | -1.4128 | 0.2117 | -6.6753 | 4.0012 | **0.0026** | -2.0004 | -0.8253 |

Table S4. Intercepts and Slopes for Random Effects (SLS)

| Level | Var | Estimate | SE | tStat | DF | pValue | Lower | Upper |
| --- | --- | --- | --- | --- | --- | --- | --- | --- |
| A | Intercept | -0.4025 | 0.2279 | -1.7659 | 4.1749 | 0.1492 | -1.0250 | 0.2200 |
| A | Squirt | 0.1398 | 0.1371 | 1.0199 | 4.8101 | 0.3563 | -0.2168 | 0.4964 |
| A | Spray | 0.3696 | 0.2128 | 1.7368 | 4.0753 | 0.1561 | -0.2169 | 0.9561 |
| B | Intercept | -0.0695 | 0.2279 | -0.3050 | 4.1749 | 0.7750 | -0.6920 | 0.5530 |
| B | Squirt | -0.1410 | 0.1371 | -1.0285 | 4.8101 | 0.3526 | -0.4976 | 0.2156 |
| B | Spray | 0.0456 | 0.2128 | 0.2143 | 4.0753 | 0.8406 | -0.5409 | 0.6321 |
| C | Intercept | 0.7441 | 0.2279 | 3.2646 | 4.1749 | **0.0291** | 0.1216 | 1.3667 |
| C | Squirt | -0.3115 | 0.1371 | -2.2726 | 4.8101 | 0.0743 | -0.6682 | 0.0451 |
| C | Spray | -0.6891 | 0.2128 | -3.2384 | 4.0753 | **0.0309** | -1.2757 | -0.1026 |
| D | Intercept | -0.2721 | 0.2279 | -1.1937 | 4.1749 | 0.2960 | -0.8946 | 0.3504 |
| D | Squirt | 0.3127 | 0.1371 | 2.2813 | 4.8101 | 0.0735 | -0.0439 | 0.6693 |
| D | Spray | 0.2739 | 0.2128 | 1.2873 | 4.0753 | 0.2662 | -0.3126 | 0.8605 |

Table S5. Intercepts and Slopes for Fixed Effects (Triton)

|  | Var | Estimate | SE | tStat | DF | pValue | Lower | Upper |
| --- | --- | --- | --- | --- | --- | --- | --- | --- |
|  | Intercept | 0.9113 | 0.2142 | 4.2544 | 4.0000 | **0.0131** | 0.3166 | 1.5061 |
|  | Squirt | -0.3299 | 0.1081 | -3.0508 | 4.0030 | **0.0380** | -0.6300 | -0.0298 |
|  | Spray | -0.7065 | 0.1926 | -3.6684 | 4.0002 | **0.0214** | -1.2412 | -0.1718 |

Table S6. Intercepts and Slopes for Random Effects (Triton)

| Level | Var | Estimate | SE | tStat | DF | pValue | Lower | Upper |
| --- | --- | --- | --- | --- | --- | --- | --- | --- |
| A | Intercept | -0.4224 | 0.2147 | -1.9670 | 4.0389 | 0.1199 | -1.0163 | 0.1716 |
| A | Squirt | 0.2106 | 0.1071 | 1.9670 | 3.8432 | 0.1235 | -0.0915 | 0.5126 |
| A | Spray | 0.3788 | 0.1926 | 1.9670 | 3.9959 | 0.1207 | -0.1561 | 0.9136 |
| B | Intercept | -0.4166 | 0.2147 | -1.9399 | 4.0389 | 0.1237 | -1.0105 | 0.1774 |
| B | Squirt | 0.2077 | 0.1071 | 1.9399 | 3.8432 | 0.1273 | -0.0944 | 0.5097 |
| B | Spray | 0.3735 | 0.1926 | 1.9399 | 3.9959 | 0.1245 | -0.1613 | 0.9084 |
| C | Intercept | 0.3063 | 0.2147 | 1.4262 | 4.0389 | 0.2263 | -0.2877 | 0.9002 |
| C | Squirt | -0.1527 | 0.1071 | -1.4262 | 3.8432 | 0.2297 | -0.4547 | 0.1494 |
| C | Spray | -0.2746 | 0.1926 | -1.4262 | 3.9959 | 0.2270 | -0.8094 | 0.2602 |
| D | Intercept | 0.5327 | 0.2147 | 2.4807 | 4.0389 | 0.0676 | -0.0612 | 1.1266 |
| D | Squirt | -0.2656 | 0.1071 | -2.4807 | 3.8432 | 0.0707 | -0.5676 | 0.0365 |
| D | Spray | -0.4777 | 0.1926 | -2.4807 | 3.9959 | 0.0682 | -1.0125 | 0.0572 |


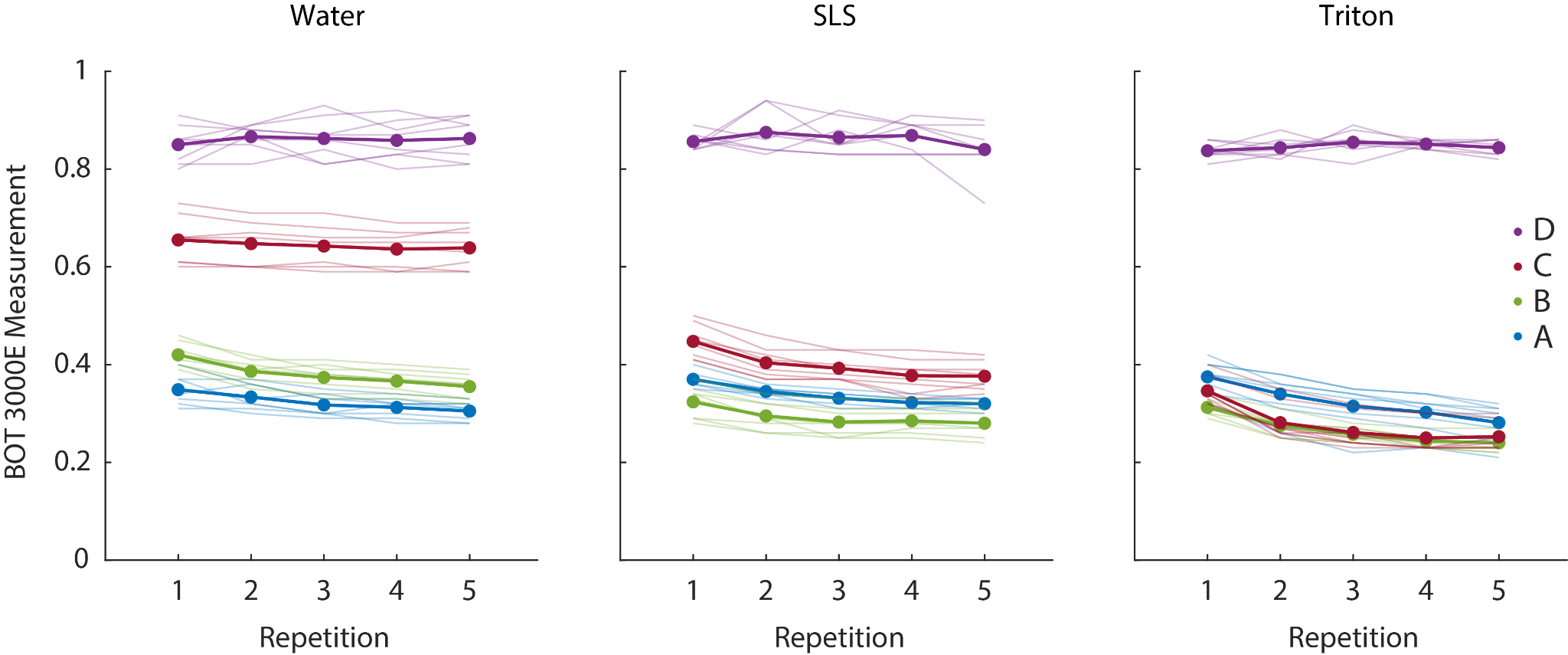


Figure S1: Test order effect for the BOT 3000E. Each series is shown as a semi-transparent line and the mean is shown as a solid line with circle markers.
